# Supplementary material for: Pyruvate Dehydrogenase Kinase 1 inhibition mediated oxidative phosphorylation enhancement in cartilage promotes osteoarthritis progression
Source: BMC Musculoskelet Disord. 2023 Jul 20;24:597. doi: 10.1186/s12891-023-06585-6 (PMC10357736; doi:10.1186/s12891-023-06585-6)
Supplement: Supplementary file 1 — Additional file 1. [file 12891_2023_6585_MOESM1_ESM.zip › KEGG.Gsea.1653622432464/KEGG_PHOSPHATIDYLINOSITOL_SIGNALING_SYSTEM.html]

Details for gene set KEGG\_PHOSPHATIDYLINOSITOL\_SIGNALING\_SYSTEM[GSEA]

|  || Dataset | 55457\_collapsed\_to\_symbols.cls.cls #OA\_versus\_normal.cls.cls #OA\_versus\_normal\_repos |
| Phenotype | cls.cls#OA\_versus\_normal\_repos |
| Upregulated in class | normal |
| GeneSet | KEGG\_PHOSPHATIDYLINOSITOL\_SIGNALING\_SYSTEM |
| Enrichment Score (ES) | -0.2557483 |
| Normalized Enrichment Score (NES) | -1.0480399 |
| Nominal p-value | 0.36925516 |
| FDR q-value | 0.5897338 |
| FWER p-Value | 1.0 |
Table: GSEA Results Summary

  

Fig 1: Enrichment plot: KEGG\_PHOSPHATIDYLINOSITOL\_SIGNALING\_SYSTEM      
 Profile of the Running ES Score & Positions of GeneSet Members on the Rank Ordered List

  

| SYMBOL | TITLE | RANK IN GENE LIST | RANK METRIC SCORE | RUNNING ES | CORE ENRICHMENT || 1 | PLCG2 | phospholipase C gamma 2 [Source:HGNC Symbol;Acc:HGNC:9066] | 725 | 0.588 | -0.0271 | No |
| 2 | PIP4K2C | phosphatidylinositol-5-phosphate 4-kinase type 2 gamma [Source:HGNC Symbol;Acc:HGNC:23786] | 939 | 0.530 | -0.0172 | No |
| 3 | CALM3 | calmodulin 3 [Source:HGNC Symbol;Acc:HGNC:1449] | 1039 | 0.510 | 0.0005 | No |
| 4 | CALML5 | calmodulin like 5 [Source:HGNC Symbol;Acc:HGNC:18180] | 1261 | 0.465 | 0.0066 | No |
| 5 | PIP5K1B | phosphatidylinositol-4-phosphate 5-kinase type 1 beta [Source:HGNC Symbol;Acc:HGNC:8995] | 1291 | 0.459 | 0.0273 | No |
| 6 | PLCE1 | phospholipase C epsilon 1 [Source:HGNC Symbol;Acc:HGNC:17175] | 1505 | 0.428 | 0.0321 | No |
| 7 | CDIPT | CDP-diacylglycerol--inositol 3-phosphatidyltransferase [Source:HGNC Symbol;Acc:HGNC:1769] | 1586 | 0.416 | 0.0466 | No |
| 8 | PIK3CB | "phosphatidylinositol-4,5-bisphosphate 3-kinase catalytic subunit beta [Source:HGNC Symbol;Acc:HGNC:8976]" | 2186 | 0.340 | 0.0169 | No |
| 9 | PLCB4 | phospholipase C beta 4 [Source:HGNC Symbol;Acc:HGNC:9059] | 2217 | 0.337 | 0.0314 | No |
| 10 | PLCD1 | phospholipase C delta 1 [Source:HGNC Symbol;Acc:HGNC:9060] | 2298 | 0.327 | 0.0415 | No |
| 11 | DGKQ | diacylglycerol kinase theta [Source:HGNC Symbol;Acc:HGNC:2856] | 2516 | 0.302 | 0.0397 | No |
| 12 | DGKI | diacylglycerol kinase iota [Source:HGNC Symbol;Acc:HGNC:2855] | 2774 | 0.273 | 0.0333 | No |
| 13 | INPP5D | inositol polyphosphate-5-phosphatase D [Source:HGNC Symbol;Acc:HGNC:6079] | 3085 | 0.241 | 0.0212 | No |
| 14 | CDS1 | CDP-diacylglycerol synthase 1 [Source:HGNC Symbol;Acc:HGNC:1800] | 3359 | 0.216 | 0.0107 | No |
| 15 | CDS2 | CDP-diacylglycerol synthase 2 [Source:HGNC Symbol;Acc:HGNC:1801] | 3361 | 0.215 | 0.0214 | No |
| 16 | PIP4K2B | phosphatidylinositol-5-phosphate 4-kinase type 2 beta [Source:HGNC Symbol;Acc:HGNC:8998] | 3416 | 0.210 | 0.0277 | No |
| 17 | ITPKB | inositol-trisphosphate 3-kinase B [Source:HGNC Symbol;Acc:HGNC:6179] | 3467 | 0.205 | 0.0340 | No |
| 18 | PLCB1 | phospholipase C beta 1 [Source:HGNC Symbol;Acc:HGNC:15917] | 3724 | 0.183 | 0.0233 | No |
| 19 | PRKCA | protein kinase C alpha [Source:HGNC Symbol;Acc:HGNC:9393] | 3728 | 0.183 | 0.0322 | No |
| 20 | ITPR2 | "inositol 1,4,5-trisphosphate receptor type 2 [Source:HGNC Symbol;Acc:HGNC:6181]" | 4222 | 0.135 | 0.0005 | No |
| 21 | PIP4K2A | phosphatidylinositol-5-phosphate 4-kinase type 2 alpha [Source:HGNC Symbol;Acc:HGNC:8997] | 4400 | 0.121 | -0.0073 | No |
| 22 | PI4KB | phosphatidylinositol 4-kinase beta [Source:HGNC Symbol;Acc:HGNC:8984] | 4485 | 0.114 | -0.0081 | No |
| 23 | INPP4A | inositol polyphosphate-4-phosphatase type I A [Source:HGNC Symbol;Acc:HGNC:6074] | 5274 | 0.050 | -0.0670 | No |
| 24 | INPP5J | inositol polyphosphate-5-phosphatase J [Source:HGNC Symbol;Acc:HGNC:8956] | 6009 | -0.005 | -0.1240 | No |
| 25 | ITPK1 | inositol-tetrakisphosphate 1-kinase [Source:HGNC Symbol;Acc:HGNC:6177] | 6095 | -0.012 | -0.1300 | No |
| 26 | INPP4B | inositol polyphosphate-4-phosphatase type II B [Source:HGNC Symbol;Acc:HGNC:6075] | 6284 | -0.026 | -0.1434 | No |
| 27 | CALML3 | calmodulin like 3 [Source:HGNC Symbol;Acc:HGNC:1452] | 6348 | -0.032 | -0.1467 | No |
| 28 | SYNJ2 | synaptojanin 2 [Source:HGNC Symbol;Acc:HGNC:11504] | 6358 | -0.032 | -0.1458 | No |
| 29 | PRKCB | protein kinase C beta [Source:HGNC Symbol;Acc:HGNC:9395] | 6453 | -0.039 | -0.1512 | No |
| 30 | PIP5K1C | phosphatidylinositol-4-phosphate 5-kinase type 1 gamma [Source:HGNC Symbol;Acc:HGNC:8996] | 6601 | -0.050 | -0.1601 | No |
| 31 | ITPR1 | "inositol 1,4,5-trisphosphate receptor type 1 [Source:HGNC Symbol;Acc:HGNC:6180]" | 6752 | -0.059 | -0.1689 | No |
| 32 | DGKB | diacylglycerol kinase beta [Source:HGNC Symbol;Acc:HGNC:2850] | 6801 | -0.063 | -0.1695 | No |
| 33 | PIK3C2B | phosphatidylinositol-4-phosphate 3-kinase catalytic subunit type 2 beta [Source:HGNC Symbol;Acc:HGNC:8972] | 6872 | -0.069 | -0.1715 | No |
| 34 | DGKG | diacylglycerol kinase gamma [Source:HGNC Symbol;Acc:HGNC:2853] | 6938 | -0.075 | -0.1728 | No |
| 35 | PLCB2 | phospholipase C beta 2 [Source:HGNC Symbol;Acc:HGNC:9055] | 7052 | -0.084 | -0.1774 | No |
| 36 | PLCB3 | phospholipase C beta 3 [Source:HGNC Symbol;Acc:HGNC:9056] | 7118 | -0.090 | -0.1780 | No |
| 37 | OCRL | OCRL inositol polyphosphate-5-phosphatase [Source:HGNC Symbol;Acc:HGNC:8108] | 7319 | -0.103 | -0.1884 | No |
| 38 | IMPA1 | inositol monophosphatase 1 [Source:HGNC Symbol;Acc:HGNC:6050] | 7332 | -0.105 | -0.1841 | No |
| 39 | PI4KA | phosphatidylinositol 4-kinase alpha [Source:HGNC Symbol;Acc:HGNC:8983] | 7397 | -0.110 | -0.1836 | No |
| 40 | PIK3CD | "phosphatidylinositol-4,5-bisphosphate 3-kinase catalytic subunit delta [Source:HGNC Symbol;Acc:HGNC:8977]" | 7405 | -0.111 | -0.1786 | No |
| 41 | DGKA | diacylglycerol kinase alpha [Source:HGNC Symbol;Acc:HGNC:2849] | 7570 | -0.125 | -0.1851 | No |
| 42 | PIK3CA | "phosphatidylinositol-4,5-bisphosphate 3-kinase catalytic subunit alpha [Source:HGNC Symbol;Acc:HGNC:8975]" | 7733 | -0.136 | -0.1909 | No |
| 43 | PIK3C2G | phosphatidylinositol-4-phosphate 3-kinase catalytic subunit type 2 gamma [Source:HGNC Symbol;Acc:HGNC:8973] | 7869 | -0.146 | -0.1942 | No |
| 44 | PIK3CG | "phosphatidylinositol-4,5-bisphosphate 3-kinase catalytic subunit gamma [Source:HGNC Symbol;Acc:HGNC:8978]" | 7953 | -0.152 | -0.1931 | No |
| 45 | INPP5K | inositol polyphosphate-5-phosphatase K [Source:HGNC Symbol;Acc:HGNC:33882] | 8103 | -0.163 | -0.1965 | No |
| 46 | PIK3C3 | phosphatidylinositol 3-kinase catalytic subunit type 3 [Source:HGNC Symbol;Acc:HGNC:8974] | 8141 | -0.166 | -0.1911 | No |
| 47 | DGKE | diacylglycerol kinase epsilon [Source:HGNC Symbol;Acc:HGNC:2852] | 8208 | -0.172 | -0.1876 | No |
| 48 | PRKCG | protein kinase C gamma [Source:HGNC Symbol;Acc:HGNC:9402] | 8582 | -0.200 | -0.2067 | No |
| 49 | PIK3R3 | phosphoinositide-3-kinase regulatory subunit 3 [Source:HGNC Symbol;Acc:HGNC:8981] | 8801 | -0.217 | -0.2129 | No |
| 50 | PIK3R5 | phosphoinositide-3-kinase regulatory subunit 5 [Source:HGNC Symbol;Acc:HGNC:30035] | 8804 | -0.217 | -0.2022 | No |
| 51 | INPP5A | inositol polyphosphate-5-phosphatase A [Source:HGNC Symbol;Acc:HGNC:6076] | 8813 | -0.218 | -0.1919 | No |
| 52 | PIK3C2A | phosphatidylinositol-4-phosphate 3-kinase catalytic subunit type 2 alpha [Source:HGNC Symbol;Acc:HGNC:8971] | 9301 | -0.256 | -0.2171 | No |
| 53 | IPPK | inositol-pentakisphosphate 2-kinase [Source:HGNC Symbol;Acc:HGNC:14645] | 9547 | -0.278 | -0.2223 | No |
| 54 | PIP5K1A | phosphatidylinositol-4-phosphate 5-kinase type 1 alpha [Source:HGNC Symbol;Acc:HGNC:8994] | 9851 | -0.306 | -0.2306 | No |
| 55 | CALM1 | calmodulin 1 [Source:HGNC Symbol;Acc:HGNC:1442] | 10136 | -0.334 | -0.2360 | No |
| 56 | ITPR3 | "inositol 1,4,5-trisphosphate receptor type 3 [Source:HGNC Symbol;Acc:HGNC:6182]" | 10390 | -0.360 | -0.2378 | Yes |
| 57 | ITPKA | inositol-trisphosphate 3-kinase A [Source:HGNC Symbol;Acc:HGNC:6178] | 10450 | -0.367 | -0.2240 | Yes |
| 58 | INPP1 | inositol polyphosphate-1-phosphatase [Source:HGNC Symbol;Acc:HGNC:6071] | 10454 | -0.367 | -0.2059 | Yes |
| 59 | DGKZ | diacylglycerol kinase zeta [Source:HGNC Symbol;Acc:HGNC:2857] | 10530 | -0.376 | -0.1929 | Yes |
| 60 | CALM2 | calmodulin 2 [Source:HGNC Symbol;Acc:HGNC:1445] | 10579 | -0.382 | -0.1775 | Yes |
| 61 | PLCG1 | phospholipase C gamma 1 [Source:HGNC Symbol;Acc:HGNC:9065] | 10722 | -0.395 | -0.1688 | Yes |
| 62 | PIK3R2 | phosphoinositide-3-kinase regulatory subunit 2 [Source:HGNC Symbol;Acc:HGNC:8980] | 10956 | -0.425 | -0.1657 | Yes |
| 63 | PIK3R1 | phosphoinositide-3-kinase regulatory subunit 1 [Source:HGNC Symbol;Acc:HGNC:8979] | 11099 | -0.443 | -0.1546 | Yes |
| 64 | SYNJ1 | synaptojanin 1 [Source:HGNC Symbol;Acc:HGNC:11503] | 11577 | -0.512 | -0.1662 | Yes |
| 65 | IMPA2 | inositol monophosphatase 2 [Source:HGNC Symbol;Acc:HGNC:6051] | 11684 | -0.530 | -0.1480 | Yes |
| 66 | PTEN | phosphatase and tensin homolog [Source:HGNC Symbol;Acc:HGNC:9588] | 11836 | -0.556 | -0.1320 | Yes |
| 67 | INPP5E | inositol polyphosphate-5-phosphatase E [Source:HGNC Symbol;Acc:HGNC:21474] | 11882 | -0.568 | -0.1071 | Yes |
| 68 | DGKD | diacylglycerol kinase delta [Source:HGNC Symbol;Acc:HGNC:2851] | 12222 | -0.651 | -0.1010 | Yes |
| 69 | INPPL1 | inositol polyphosphate phosphatase like 1 [Source:HGNC Symbol;Acc:HGNC:6080] | 12335 | -0.687 | -0.0753 | Yes |
| 70 | PIKFYVE | "phosphoinositide kinase, FYVE-type zinc finger containing [Source:HGNC Symbol;Acc:HGNC:23785]" | 12347 | -0.690 | -0.0417 | Yes |
| 71 | DGKH | diacylglycerol kinase eta [Source:HGNC Symbol;Acc:HGNC:2854] | 12619 | -0.809 | -0.0224 | Yes |
| 72 | INPP5B | inositol polyphosphate-5-phosphatase B [Source:HGNC Symbol;Acc:HGNC:6077] | 12723 | -0.884 | 0.0138 | Yes |
Table: GSEA details [plain text format]

  

Fig 2: KEGG\_PHOSPHATIDYLINOSITOL\_SIGNALING\_SYSTEM      
 Blue-Pink O' Gram in the Space of the Analyzed GeneSet

  

Fig 3: KEGG\_PHOSPHATIDYLINOSITOL\_SIGNALING\_SYSTEM: Random ES distribution      
 Gene set null distribution of ES for **KEGG\_PHOSPHATIDYLINOSITOL\_SIGNALING\_SYSTEM**
